# A Manual of Cystoscopy

**Published:** 1911-07

**Authors:** 


**A Manual of Cystoscopy.** By **J. Bently Squier,** M.D., Professor of Genitourinary Surgery, New York Post-Graduate Medical School and Hospital, and **Henry G. Bugbee,** M.D., Instructor in Genitourinary Surgery, New York Post-Graduate Medical School and Hospital. 12 mo, pp. 117, with twenty-six original plates. New York: Paul B. Hoeber. 1911. (Flexible leather, $3.00 net.)

Examining this book one is struck by its simplicity of language, its clarity of description and its general excellence of construction. It bears the mark of careful preparation and the illustrations are helpful for the very simple reason that they give the observer, even though he has never looked within a human urinary bladder, a very definite and accurate idea of what the interior of the organ is like in normal and pathologic conditions. In the preface it is stated that the book was written in response to student demands for a practical work on cystoscopy; a book that would be useful and instructive to one unacquainted with the technical side of cystoscopy. How well it fills this mission may be gathered when it is stated without any reservation that it is by all odds the best book on cystoscopy in compact form that has ever been issued and more real working information may be secured from its pages than from many of the more pretentious efforts toward literary cystoscopic expedition. A book barren of error is almost impossible of production, strange as it may seem, yet it appears rather careless on the part of the publisher to permit the statement that the colored plates of "intravesicle" lesions are particularly accurate. It is, of course, apparent that "intravesical" was meant.

The history of cystoscopy is sketched from its beginning in 1807, when candles were used as the illuminating medium, up to the present time of perfection of technic and instruments. The manner of manipulation of the direct and indirect instruments is minutely described and the advantages of each are set forth clearly as are their disadvantages in certain phases of examination. An ingenious description is that of the making of a home-made phantom bladder which will be as useful for cystoscopic practice as most of the very high priced and complicated phantoms which are offered to enthusiasts for the betterment of their skill.

It is not flattery to say that one wholly unacquainted with any of the detail of cystoscopic manipulation can with fair measure of success after a careful reading of this book make a satisfactory examination and reading of a bladder. It will not require more than average human intelligence to recognise the conditions described by Squier and illustrated by his artist in colored plates, for while the pictures lack that brilliancy of coloring and that clarity of perspective which one sees through the cystoscope in pathologic and normal conditions, the general impression is there and the drawings are far above the average illustration of pathologic condition in point of artistic merit. The artist has caught the salient point in every case and has emphasised it with gentle skill. The flatness of the views is undoubtedly due to the use of the screen process of reproduction. Aside from this purely technical deficiency the illustrations are excellent. Particularly true are those plates which illustrate the tubercular bladder. They are without flaw.

The main value of the book, however, lies in its excellent description of how to make a cystoscopic examination and what one will see in normal and pathologic conditions. There could hardly be any error of consequence in the use of this most essential instrument if directions are followed. No detail, however insignificant apparently, is omitted in describing how the instrument should be inserted and how turned and elevated and depressed to get the best results. No writer on cystoscopy has yet attained the perfection in description of manipulation that is found in this book and it takes rank as one of the gems of literary excellence and instructive writing. It is a simple, direct and excellent book wholly lacking in high-browed and egotistic enthusiasm. That, added to its intrinsic value, makes it invaluable.

